# Entomologic and Virologic Investigation of Chikungunya, Singapore

**DOI:** 10.3201/eid1508.081486

**Published:** 2009-08

**Authors:** Lee-Ching Ng, Li-Kiang Tan, Cheong-Huat Tan, Sharon S.Y. Tan, Hapuarachchige C. Hapuarachchi, Kwoon-Yong Pok, Yee-Ling Lai, Sai-Gek Lam-Phua, Göran Bucht, Raymond T.P. Lin, Yee-Sin Leo, Boon-Hian Tan, Hwi-Kwang Han, Peng-Lim S Ooi, Lyn James, Seow-Poh Khoo

**Affiliations:** National Environment Agency, Singapore (L.-C. Ng, L.-K. Tan, C.-H. Tan, S.S.Y. Tan, H.C. Hapuarachchi, K.-Y. Pok, Y.-L. Lai, S.-G. Lam-Phua, G. Bucht, S.-P. Khoo); Tan Tock Seng Hospital, Singapore (R.T.P. Lin, Y.-S. Leo); Ministry of Health, Singapore (B.-H. Tan, H.-K. Han, P.-L. S. Ooi, L. James)

**Keywords:** Chikungunya, chikungunya virus, surveillance, entomology, virology, sequencing, phylogenetic analysis, Singapore, viruses, research

## Abstract

Local transmission of chikungunya, a debilitating mosquito-borne viral disease, was first reported in Singapore in January 2008. After 3 months of absence, locally acquired Chikungunya cases resurfaced in May 2008, causing an outbreak that resulted in a total of 231 cases by September 2008. The circulating viruses were related to East, Central, and South African genotypes that emerged in the Indian Ocean region in 2005. The first local outbreak was due to a wild-type virus (alanine at codon 226 of the envelope 1 gene) and occurred in an area where *Aedes aegypti* mosquitoes were the primary vector. Strains isolated during subsequent outbreaks showed alanine to valine substitution (A226V) and largely spread in areas predominated by *Ae. albopictus* mosquitoes*.* These findings led to a revision of the current vector control strategy in Singapore. This report highlights the use of entomologic and virologic data to assist in the control of chikungunya in disease-endemic areas.

## Abstract 2

Data from longitudinal analyses can be useful in the design and implementation of control strategies.

Chikungunya is a mosquito-borne infectious disease caused by chikungunya virus (CHIKV), which belongs to the family *Togaviridae* and genus *Alphavirus*. CHIKV causes a nonfatal, self-limiting disease characterized by abrupt onset of high fever, severe arthralgia, or arthritis, often associated with skin rash.

CHIKV was first isolated during an outbreak in Tanganyika (now Tanzania) in 1952–1953 (*1*). The virus is believed to have originated in Africa and subsequently was introduced into many regions of Asia (*2*). The first CHIKV isolation in Asia was in Thailand in 1958 (*3*), followed by India in 1963 (*4*). A 2002–2003 serosurvey on 531 healthy young adults in Singapore showed a low prevalence (0.3%) of chikungunya antibodies (*5*). Although CHIKV has caused several large-scale epidemics in Asia and the Pacific region, it largely was neglected until its reemergence in the Indian Ocean Islands in early 2005 (*6*). Since then, CHIKV has caused outbreaks in India (*7*), Sri Lanka (*8*), Singapore (*9*), Malaysia (*10*), and Italy (*11*), focusing global attention on this newly emerging disease.

CHIKV is an enveloped, positive strand RNA virus with a genome of ≈11.8 kb (*12*). Phylogenetic analysis of the CHIKV genome has identified 3 lineages; West African, Asian and East, and Central and South African (ECSA) (*13*). The Asian lineage circulated in Asia until it was replaced by the ECSA type, which emerged during the 2005–2006 outbreaks in the Indian Ocean Islands and India (*6*).

Unlike in Africa, where the virus is maintained in a sylvatic cycle, chikungunya in Asia has been an urban disease, typically found in dengue-endemic areas and transmitted largely by *Aedes aegypti* mosquitoes. However, the predominant *Aedes* sp. in locations such as Réunion Island, where chikungunya emerged in 2005, was *Ae. albopictus* (*14*). The spread of chikungunya into rural areas during the later stages of outbreaks in India further confirmed the potential of *Ae. albopictus* mosquitoes in transmitting CHIKV (*15*). These changes were concurrent with the emergence of a strain having an alanine to valine substitution at codon 226 (A226V) of the envelope 1 (*E1*) gene in Réunion Island (*16*) and India (*17*). This mutation is known to increase the transmissibility of the virus by *Ae. albopictus* mosquitoes (*18*).

Because there is no licensed vaccine or specific drug therapy available to cure the illness, intervention relies upon vector control and minimizing mosquito-human contact. The first chikungunya outbreak in Singapore during January 2008 was successfully contained by combining aggressive vector control operations with active case detection and isolation of patients (*9*). On February 21, 2008, 24 days (2 incubation periods) after the last reported case, the outbreak was declared closed (*9*). After 3 months of no cases, local chikungunya cases resurfaced in May 2008 causing an outbreak that is yet to be resolved. This outbreak coincided with a rise in chikungunya incidence in Malaysia (*10*). In this report, we focus on the virologic and entomologic investigations carried out in Singapore, which assisted in the effort against the emergence of chikungunya in 2008.

## Methods

### Case Surveillance

Singapore initiated a chikungunya surveillance system in late 2006. The medical community was apprised by the Ministry of Health to look out for chikungunya cases among febrile patients, especially when associated with symptoms and signs (e.g., arthralgia, rash) suggestive of chikungunya (*9*). At the Environmental Health Institute (EHI), a national public health laboratory, an active laboratory-based surveillance was set up among a network of general practitioners. Confirmed cases were categorized as imported or local based on detailed travel history. Virologic analysis described in this study was performed on samples received by the EHI as part of the national public health surveillance program designed for chikungunya in Singapore.

### Laboratory Diagnosis

Diagnosis of chikungunya was confirmed by detection of a fragment of the nonstructural protein 1 gene of CHIKV by a real-time reverse transcription–PCR (RT-PCR) protocol described previously (*19*). CHIKV RNA was extracted from serum by using QIAamp viral RNA mini kit (QIAGEN, Hilden, Germany), and the amplification was performed in a LightCycler 2.0 system by using LightCycler RNA Master SYBR Green I kit (Roche Diagnostics GMbH, Mannheim, Germany) according to manufacturers’ instructions. All tests included 2 negative controls: a PCR control and a negative extraction control of DNAse/RNAse-free water. The positive control was RNA extracted from a CHIKV culture with a known PFU titer determined by plaque assay. The presence of CHIKV was determined based on the melting peaks (83.07°C–84.17°C) of the positive control amplifications.

### Design of Specific Primers for Sequencing

All primers were essentially constructed towards strains of the Indian Ocean and Central African origin using Gene Runner 3.05 (Hastings Software, Inc., Hastings, NY, USA) and Primer Select 5.03 (DNASTAR Inc., Madison, WI, USA) software. The primer sequences used are listed in Table 1.

**Table 1:** Primers for DNA template synthesis and sequencing of chikungunya virus, Singapore*

Name/genomic position†	Sequence (5′ → 3′)
ChikE1/9870F	ACAAGCCCTTATTCCGCTG
ChikE1/9994F	TACGAACACGTAACAGTGATC
ChikE1/10246F	TACCCATTTATGTGGGGC
ChikE1/10378F	GCATCAGCTAAGCTCCGC
ChikE1/10397R	ACGCGGAGCTTAGCTGAT
ChikE1/10521R	ACCTTTGTACACCACAATT
ChikE1/10643F	CACAACTGGTACTGCAGAGACC
ChikE1/10710R	GCCAGATGGTGCCTGAGA
ChikE1/10965F	GAAAGGCAAGTGTGCGGT
ChikE1/10993R	TCATCGAATGCACCGCAC
ChikE1/11232F	CACGGGAGGTGTGGGAC
ChikE1/11238R	TCCCGTGATCTTCTGCACC
ChikE1/11359R	GTGTGTCTCTTAGGGGACACATA

*Chik, chikungunya; F, forward primer; R, reverse primer. †Genomic position of chikungunya virus (GenBank accession no. DQ443544.2) to which the first base (5′ end) of the primer corresponds.

### Sequencing of the E1 Gene

Complimentary DNA was synthesized as described in SuperScript III First-Strand synthesis system for RT-PCR (Invitrogen Corp., Carlsbad, CA, USA). All templates were purified with the QIAquick PCR purification kit (QIAGEN) before sequencing. Sequencing was performed using BigDye Terminator Cycle Sequencing kit, according to manufacturer’s instructions (Applied Biosystems, Foster City, CA, USA).

### Phylogenetic Analysis

The nucleotide sequences were assembled using the SeqMan II version 5.03 (DNASTAR) and aligned using Clustal W multiple alignment tool in the BioEdit Sequence Alignment Editor version 7.0.9.0 (*20*). The phylogenetic tree was inferred based on the 1,002-nt sequence of the *E1* gene from aa residues 91 to 424, using the maximum-likelihood (ML) method as implemented in PAUP* version 4.0b10 (*21*). Bootstrapping to access the robustness of the ML tree topology was performed using the neighbor-joining method under the ML criterion based on 1,000 replicates.

### Entomologic Surveillance

**Figure 1:**
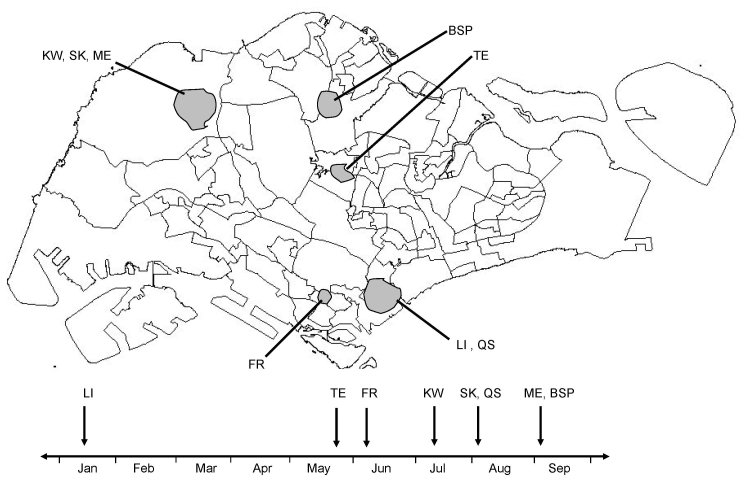
Geographic and temporal distribution of 123 indigenous chikungunya cases in Singapore. Shading indicates the 7 cluster areas where entomologic investigation was carried out. Data include cases reported through September 2008. The arrows in the timeline shown below the map indicate the months of occurrence of the local outbreaks from the beginning of January to the end of September 2008. BSP, Bah Soon Pah Road; FR, Farrer Road; KW, Kranji Way; LI, Little India; ME, Mandai Estate; QS, Queen Street; SK, Sungei Kadut; TE, Teachers’ Estate.

Seven local transmission clusters representing major local outbreaks were selected for entomologic investigation: Little India (1°18′24′′N, 103°50′57′′E), Queen Street (1°17′ 52′′N, 103°51′05′′E), Teachers’ Estate (1°23′0′′N, 103° 49′43′′E), Kranji (1°25′30′′N, 103°45′43′′E), Sungei Kadut (1°25′1′′N, 103°45′2′′E), Mandai Estate (1°24′31′′N, 103°45′34′′E), and Bah Soon Pah Road (1° 24′45′′N, 103°49′E) (Figure 1). These areas were classified naturally into urban (Little India and Queen Street), suburban (Teachers’ Estate) and rural (Kranji, Sungei Kadut, Mandai Estate and Bah Soon Pah Road). The georeferenced *Aedes* larvae collection data from the chikungunya clusters were extracted from the Geographic Information System (ArcGIS) database of the National Environmental Agency, Singapore. The database, which is a part of the national vector control program, was assembled based on routine vector surveillance data obtained daily through area-wide inspection for mosquito breeding by ≈500 vector control officers.

The ultimate objective of this routine exercise was to identify as many active breeding places as possible in all residential and nonresidential premises within each cluster area. The collected larvae were separated into species based on morphologic identification before their numbers were counted. For this study, larval surveillance data were expressed as the larval abundance index, the ratio between the numbers of *Ae. aegypti* and *Ae. albopictus* larvae collected. For a single case, the number of larvae found within a 200-m radius of the case was used to calculate the larval abundance index, whereas the number of larvae found within the boundary of the cluster area was used in widespread clusters. Larval data collected 3 months before and after the first case reported from each cluster were used to calculate the index.

In each cluster area selected, adult mosquito surveillance was also conducted to determine the *Aedes* spp. composition and to confirm the presence of CHIKV in identified mosquitoes. Adult mosquitoes were collected using the sweep-net method, the Biogents (BG) Sentinel Trap (Biogents AG, Regensburg, Germany) or both. In each area, adult mosquito surveillance was conducted within 1-week from the beginning of the outbreaks, usually at the location from where the highest number of cases was reported. The survey was conducted once in all areas, except for Kranji Way, where it was carried out twice with a gap of 1 week between each collection. The number of locations surveyed ranged from 1 to 25 premises in each area, with higher number of premises in urban areas and lower numbers in rural areas in general. However, if a single case was reported from a cluster area, the adult mosquito survey was conducted in a few randomly selected premises within the neighboring area of the index case, even if it was an urban area. The sweep net method was performed in Little India and Teachers’ Estate areas. The BG Sentinel traps were deployed in Queen Street, Sungei Kadut, Mandai Estate, and Bah Soon Pah Road areas. The number of traps deployed in each area ranged from 4 to 15 traps, with a trapping duration of 12 to 24 hours on each occasion. The sweep net method and BG Sentinel traps were used in Kranji Way. Adult *Aedes* mosquitoes were crushed individually in minimum essential medium before RT-PCR was performed as for serum samples. The isolated viruses were sequenced and analyzed as described above.

## Results

### Chikungunya Cases

From December 2006 through December 2007, a total of 1,375 samples were tested at the EHI for chikungunya; 10 of these cases were positive by PCR or immunoglobulin M testing. Epidemiologic investigation showed that all these cases were imported from India, Maldives, Sri Lanka, and Indonesia, which generally reflected the regional distribution of chikungunya during that time.

More than 7,000 samples from general practitioners, hospitals, and active case detection were tested from January through September 2008. In January 2008, the first locally acquired case of chikungunya was detected in the Little India area by a general practitioner involved in the chikungunya surveillance network (Figure 1). A total of 13 locally acquired chikungunya cases were confirmed by PCR before the outbreak was finally brought under control.

Between the first episode of transmission and May 2008, 6 cases imported from Sri Lanka (n = 2), Indonesia (n = 3), and Malaysia (n = 1) were diagnosed. By June, the number of imported cases increased, and the local scene remained relatively quiet with only 2 episodes of local transmission in Teachers’ Estate area in late May (2 cases) and Farrer Road area in early June (1 case) (Figure 1). Both of these episodes were in suburban residential areas. Active case detection did not show any additional cases associated with those 2 episodes. Locally acquired cases occurred again in July 2008 coinciding with a rise in imported cases from Malaysia. By the end of September 2008, there was a cumulative total of 231 cases comprising 108 imported and 123 locally acquired infections. Of the imported cases, 92% (n = 99) had travel history to Malaysia, largely to the state of Johor, whereas the 123 local cases were distributed across 25 different locations.

After July 2008, transmission was more active in rural industrial and farming areas of Singapore, with the biggest clusters being in Kranji, Sungei Kadut, and Bah Soon Pah Road (Figure 1). Notably, during the active case surveillance using PCR, 2 viremic cases were found 1 day before the onset of clinical manifestations, with viral loads of 750 pfu/mL and 40 pfu/mL of blood, determined by using an external standard curve generated by plotting 10-fold serially diluted virus from a concentration of 10^8^ pfu/mL, against respective crossing-point values of real-time PCRs.

### Virologic Investigation

**Figure 2:**
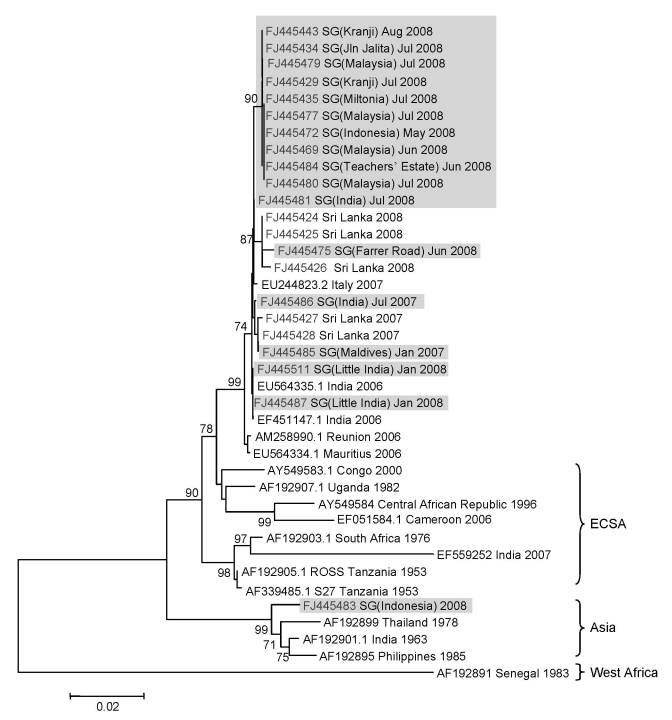
Phylogenetic analysis of the chikungunya virus (CHIKV) envelope 1 (*E1*) gene. The maximum-likelihood method was used to construct the phylogenetic tree by using 1,002 nucleotides of the sequence of the *E1* gene from codons 91 to 424. The tree included 17 isolates detected in Singapore (shaded), 5 Sri Lankan isolates sequenced at the Environmental Health Institute, and 17 global sequences selected to represent all known phylogenetic lineages. In the tree, all sequences are labeled with GenBank accession numbers and country of origin, and are isolated by year/month. In addition, all locally acquired and imported Singapore isolates are labeled with the reported area and country of origin, respectively, within parentheses. Only the bootstrap values >70 are shown on branches. Scale bar indicates nucleotide substitutions per site. ECSA, East, Central and South African genotype; SG, Singapore.

The *E1* gene of CHIKV from 85 imported and locally acquired infections was analyzed. Because there were several groups of similar sequences, the phylogenetic tree was constructed by using only 17 sequences that represented all imported as well as locally acquired strains at different time points. The tree also included 5 CHIKV from Sri Lanka sequenced at the EHI and 17 global sequences retrieved from the GenBank database (Figure 2). Phylogenetic analysis showed that all viruses reported in Singapore after January 2008, except 1, were related to the ECSA genotype. CHIKV isolated from the remaining infection was of Asian lineage and was imported from Indonesia (Figure 2). All ECSA-type viruses formed a distinct clade, together with isolates from India, Sri Lanka, Italy, and the Indian Ocean Islands (Figure 2). In the phylogenetic tree, the viruses isolated during the first outbreak in the Little India area clustered closely with those reported in India in 2006. One isolate from an imported case from Maldives also clustered within this group. In contrast, viruses isolated during the second local episode in the Teachers’ Estate in May 2008 and in all other areas from July 2008 grouped with those imported from Malaysia. Similarly, CHIKV isolated during the third local episode in the Farrer Road area in June 2008 clustered separately with isolates from Sri Lanka (Figure 2).

CHIKV isolated during the first local outbreak was wild-type (alanine) at aa residue 226 (A226) of the *E1* gene, whereas, those detected during the second, third, and subsequent local episodes contained valine (A226V). Besides A226V, CHIKV isolated during the second local outbreak showed 2 synonymous mutations at nucleotide positions 300 (C300T) and 363 (A363G) of the *E1* gene, which were not present in viruses involved in the first and third outbreaks. Of these isolates, C300T was unique to CHIKV strains imported from Malaysia. C300T and A363G were also found in all viruses detected in imported cases from Malaysia after June 2008. Similarly, CHIKV isolated in the third local episode was unique because it showed 2 synonymous mutations at nucleotide positions 105 (A105G), 1308 (C1308T) and a nonsynonymous mutation at nucleotide position 633 (A633C [K211N]) of the *E1* gene, the combination of which was unique to CHIKV isolates from Sri Lanka. Therefore, we defined the combinations of C300T + A363G and A105G + A633C + C1308T as genetic signatures of isolates from Malaysia and Sri Lanka, respectively. These observations demonstrated that the first 3 episodes of chikungunya transmission in Singapore were most likely due to independent importations of distinct viruses from different geographic locations.

### Entomologic Investigation

*Aedes* larval collection data showed that *Ae. albopictus* was the predominant species in all cluster areas, except Little India, an urban area where the first outbreak occurred (Table 2). In the Little India cluster, larval abundance index in the Clive Street area (2.14:1) was even higher than the generalized ratio for the whole cluster (1.77:1). The Clive Street area is a highly urbanized area and reported the highest number of chikungunya cases (n = 10) within the Little India cluster. This observation was further strengthened by adult mosquito surveillance, which yielded only *Ae. aegypti* in the Little India cluster*.* In contrast, *Ae. albopictus* (n = 164) was the only *Aedes* sp. caught in other cluster areas (Table 2). Adult *Ae. albopictus* mosquitoes from the Kranji Way and Bah Soon Pah Road areas were positive for CHIKV by RT-PCR. In Kranji Way, 7 (9.1%) of 77 female *Ae. albopictus* mosquitoes were positive for CHIKV, whereas 6 (13.5%) of 45 mosquitoes were positive in the Bah Soon Pah Road area. The *E1* gene sequences of those 13 *Ae. albopitcus*-borne CHIKV were identical to sequences of strains imported from Malaysia. All mosquito-borne viruses possessed the A226V substitution.

**Table 2:** Summary of the characteristics and entomologic data of chikungunya cluster areas, Singapore

Location	Type	No. cases*	Adult female mosquito collection†		*Aedes* larval abundance index‡
			*Aedes aegypti*	*Ae. albopictus*	
Little India	Urban	13	10	0	1.77:1 (826:466)
Queen Street	Urban	1	0	2	0:1 (0:127)
Teachers’ Estate	Suburban	1	0	10	0.03:1 (40:1,261)
Kranji Way	Rural	41	0	77	0.04:1 (1,129:26,546)
Sungei Kadut	Rural	33	0	7	0.001:1 (70:77,086)
Mandai Estate	Rural	11	0	23	0.02:1 (30:1,260)
Bah Soon Pah Road	Rural	21	0	45	0:1 (0:3,465)

*Numbers are preliminary data from press releases. †Species of adult mosquitoes collected in each location where entomologic surveillance was conducted. The numbers do not necessarily represent adult mosquito density in each area as the numbers of traps and man-hours committed were not consistent. ‡*Aedes* larval abundance index is expressed as the ratio between the number of *Ae. aegypti* and *Ae. albopictus* larvae collected through routine surveillance, 3 months before and up to 3 months after the detection of the first case at respective locations. Number of larvae (*Ae. aegypti; Ae. albopictus*) collected in each cluster is shown in parentheses.

## Discussion

Chikungunya is an emerging infectious disease of public health importance in Singapore. Owing to Singapore’s small size, tropical climate, presence of the vectors, and high population density, timely and effective disease control is required to minimize the risk for chikungunya outbreaks. Since its emergence on the local scene in January 2008, entomologic and virologic investigations have been used to elucidate the origin of the current outbreak of chikungunya in Singapore.

Phylogenetic data showed that the first, second, and third episodes of local transmission from January 14, 2008 to June 9, 2008, were due to 3 genetically distinct viruses of different geographic origins. The first outbreak in the Little India area in January 2008 was due to a CHIKV strain of Indian origin, whereas the second episode (2 cases) in Teachers’ Estate area in May was due to a strain closely related to viruses detected in cases imported from Malaysia. On the other hand, the CHIKV strain of the third episode (1 case) in Farrer Road area in June was closely related to isolates from Sri Lanka. According to epidemiologic data, no locally acquired chikungunya cases occurred between the first and the second episodes. Similarly, no cases were reported between the second and third episodes. Therefore, the possibility that CHIKV involved in the first outbreak evolved into genetically distinct strains detected in the second and third episodes was highly unlikely.

The unique genetic signatures among these viruses and the lack of local transmission between episodes indicated that the first 3 local episodes were most likely due to independent importations of CHIKV, most likely from India, Malaysia, and Sri Lanka. This finding was further supported by the fact that 6 imported cases reported during the first and second episodes included cases imported from Malaysia and 2 from Sri Lanka. However, all cases reported after July 2008 were due to a single strain, which was closely related to CHIKV detected in cases imported from Malaysia. This strain was genetically close to the virus that caused the second episode. Recently, it was reported that the 2007 chikungunya outbreak in Malaysia was due to a virus of the ECSA lineage (*10*). This evidence points to the interconnectedness of simultaneous chikungunya outbreaks in Singapore and Malaysia, which is not unexpected given the close proximity and porous borders between these 2 countries.

Entomologic surveillance showed a difference between the vector species involved in the first and subsequent outbreaks in Singapore. All adult mosquitoes caught in the vicinity of the first outbreak area (Little India) were *Ae. aegypti*. The larval surveillance data also showed the predominance of *Ae. aegypti* mosquitoes in this area (Table 2). Little India is generally a highly urbanized area with sparse vegetation, which could explain the presence of more *Ae. aegypti* vectors than *Ae. albopictus*. On the other hand, subsequent chikungunya episodes were seen in less-urbanized areas (Table 2) where *Ae. albopictus* was the predominant vector species. Detection of CHIKV in *Ae. albopictus* mosquitoes further confirmed its role in CHIKV transmission in less urbanized areas. In general, large clusters of chikungunya were seen in less urbanized areas, with a high *Ae. albopictus* mosquito density near human habitations.

Of note, CHIKV strains isolated from Little India, an *Ae. aegypti* mosquito–abundant area, showed alanine at codon 226 (A226) of the *E1* gene. In contrast, all CHIKV strains isolated during subsequent episodes showed A226V substitution and were distributed in areas that were mainly inhabited by *Ae. albopictus* mosquitoes. Recently, Tsetsarkin et al. showed that CHIKV strains with A226V substitution replicate better in *Ae. albopictus* mosquitoes than does the wild-type strain (*18*). Their findings indicated that although the transmission potential of the wild-type virus is optimum for *Ae. aegypti* mosquitoes, A226V substitution confers greater vector competence in *Ae. albopictus* mosquitoes, making the latter species a better vector of the mutated strain than *Ae. aegypti* (*18*). This finding may result in selection for the mutated strain in areas where *Ae. albopictus* mosquitoes are abundant. Although the competence of *Ae. aegypti* mosquitoes in transmitting the virus with A226V in Singapore remains uncertain, the known evidence may therefore explain why the mutant virus with A226V caused outbreaks in less urbanized areas in Singapore where *Ae. albopictus* mosquitoes dominate but had little effect on urbanized areas where *Ae. aegypti* mosquitoes dominate. Similarly, this finding could also explain the distribution of the wild-type (A226) strain in urban areas, where *Ae. aegypti* mosquitoes are predominantly found. A similar observation has also been made in India, where the emergence of CHIKV with A226V was first reported in rural areas of Kerala region that are predominantly inhabited by *Ae. albopictus* mosquitoes (*15*). The low transmission rate of the mutant virus in urban and suburban Singapore could also be due to the aggressive dengue control program, which targets mainly urban and suburban parts of the country.

Based on these observations, the National Environment Agency’s *Aedes* spp. control strategy was revised and expanded, especially in areas where *Ae. albopictus* mosquitoes are present. Because *Ae. albopictus* are generally outdoor mosquitoes, in contrast to *Ae. aegypti*, measures such as outdoor fogging and residual spray of external walls were conducted in chikungunya outbreak areas. The phylogenetic data was mainly used to trace the possible origins of viral strains causing the local chikungunya episodes. The longitudinal monitoring of *E1* gene sequences of CHIKV is in progress to monitor local transmission of chikungunya in Singapore. Our results showed that Singapore, being a travel hub and a cosmopolitan city, is vulnerable to multiple importations of CHIKV. The aggressive A226V variant of the ECSA genotype that has established itself in the region is posing a challenge to Singapore. Because *Ae. albopictus* is a common vector species in the region, the establishment of the A266V CHIKV variant in the region may continue to pose challenges in the years to come.
